# Pregnancy loss in rural Bangladesh: an analysis of rates, proportions, timing, and determinants based on data from a Health and Demographic Surveillance System

**DOI:** 10.7189/jogh.16.04101

**Published:** 2026-03-27

**Authors:** Sahar Raza, Rajon Banik, Syed Toukir Ahmed Noor, Qazi Sadeq-ur Rahman, Farha Nusrat Zahan, Md Abu Bakkar Siddique, Md Mehedi Hasan, Tamanna Majid, Esrat Jahan, Abu Sayeed, Lubna Hossain, Saraban Tahura Ether, Afruna Rahman, Nafisa Huq, Shams El Arifeen, Anisuddin Ahmed, Ahmed Ehsanur Rahman

**Affiliations:** 1 Maternal and Child Health Division, International Centre for Diarrhoeal Disease Research, Bangladesh (icddr,b), Dhaka, Bangladesh; 2 Faculty of Health, Medicine and Behavioural Sciences, The University of Queensland, St Lucia, Queensland, Australia; 3 Infectious Diseases Division, International Centre for Diarrhoeal Disease Research, Bangladesh (icddr,b), Dhaka, Bangladesh; 4 Independent University, Bangladesh (IUB), Dhaka, Bangladesh

## Abstract

**Background:**

Pregnancy loss, encompassing miscarriage/spontaneous and induced abortion, has substantial physical and mental health consequences for women. However, population-level evidence on its occurrence in Bangladesh remains limited, with most data derived from reports and small-scale community-based studies. Here we provide population-level estimates of pregnancy loss and examine associated factors in rural Bangladesh.

**Methods:**

We analysed data from 61 428 women aged 15–49 years and 7612 pregnancies, extracted from the Baliakandi Health and Demographic Surveillance System of icddr,b and covering the period from 1 August 2021 to 30 June 2023. We used descriptive statistics with 95% confidence intervals (CIs) to report rates and proportions, and a generalised linear mixed model to identify factors associated with pregnancy loss.

**Results:**

Overall, the rate of pregnancy loss was 13.9 (95% CI = 13.2–14.6) per 1000 women per year, while the proportion was 14% of all recognised pregnancies. Around 11% of pregnancies resulted in miscarriage/spontaneous abortion and 3% in induced abortion. Miscarriage/spontaneous abortions peaked between 11 and 13 weeks, and induced abortions between 6 and 7 weeks of gestation. Women with a prior history of pregnancy loss or stillbirth had higher odds of miscarriage/spontaneous abortion. Higher maternal age was associated with both miscarriage/spontaneous abortion and induced abortion, while induced abortion was associated with women with larger family sizes.

**Conclusions:**

Pregnancy loss is notably prevalent in rural Bangladesh, predominantly occurring spontaneously and during the first trimester. Maternal health initiatives should prioritise closer monitoring and support during the early stages of pregnancy, particularly for women with a history of pregnancy loss.

The term ‘pregnancy loss’ is defined by intrauterine foetal demise before the viable age of gestation [1], which can range from 20 to 28 weeks of pregnancy, depending on geographical region [2]. It can bring significant physical, psychological, and financial burden to the woman, regardless of whether it is unintentional (miscarriage/spontaneous abortion) or intentional (induced abortion) [2]. According to the World Health Organization (WHO), a miscarriage/spontaneous abortion is the spontaneous loss of pregnancy before 22 completed weeks of gestation, while an induced abortion is the intentional termination of intrauterine pregnancy before 20–24 completed weeks of gestation [2].

When a woman loses her pregnancy, she may face physical complications such as bleeding, infections, and internal organ damage [3,^5^]. These risks become greater if the procedure is performed in an unsafe manner [6] and may lead to long-term consequences such as arrhythmia, cardiovascular disease, premature birth, and infertility [7,8]. Pregnancy loss also raises the risk of posttraumatic stress disorder, anxiety, depression, and, in severe cases, suicide [9,10], while the associated shame and stigma lead to the concealment of ensuing physical and mental symptoms [11]. Pregnancy loss can also create a significant financial burden, with Global Burden of Disease (GBD) estimates indicating approximately 1.3 million disability-adjusted life years lost globally [12].

According to a 2021 Lancet Series, an estimated 23 million miscarriages occur every year globally, with 15% of all documented pregnancies resulting in miscarriage [3]. Additionally, an estimated 39 induced abortions per 1000 women (aged 15–45 years) per year occur globally, equating to 73 million induced abortions annually [13]. A study conducted across 33 low- and middle-income countries found that South Asia accounted for the highest proportion of pregnancy loss cases (43.4%), followed by sub-Saharan Africa (27.8%) and South America (12.6%) [14]. Similarly, research has indicated that India, China, Ethiopia, and Pakistan were the four countries with the highest occurrence of pregnancy loss, accounting for 38% of the global incidence [15].

A recent study from a coastal region of Bangladesh found that approximately 11% of pregnancies resulted in miscarriage, and 4% of all recognised pregnancies ended in induced abortion [16]. Furthermore, an estimated 1.2 million induced abortions were performed in Bangladesh, with a rate of 29 per 1000 women (aged 15–49) in 2014 [17]. According to the 2020 report of the Health and Demographic Surveillance System (HDSS) in Matlab, Bangladesh, 457 (~8%) of 6136 pregnancies were miscarriages and 91 (~2%) were induced abortions [18].

In this context, pregnancy loss has been set as a global agenda, with universal access to sexual and reproductive health by 2030 being a part of the Sustainable Development Goals [19] and the worldwide strategy for women’s, children’s, and adolescents’ health [20]. Updated estimates of the pregnancy loss are therefore key for informing policy or practice that would safeguard these vulnerable women.

However, significant gaps remain in our understanding of the population-level burden of pregnancy loss in Bangladesh, as available evidence is largely derived from reports and small-scale community-based studies [16,18]. This is particularly true for comprehensive and up-to-date estimates that include both miscarriage/spontaneous abortion and induced abortion using consistent denominators. Existing evidence reports on miscarriage primarily among pregnant women, while induced abortions are estimated indirectly among women of reproductive age, limiting comparability and robust assessment [16,17]. This fragmented approach constrains our understanding of the overall burden of pregnancy loss, including its timing by gestational age and associated social, demographic, and health determinants. Direct, population-level estimates using unified denominators are therefore needed to inform targeted interventions, particularly for miscarriage and early pregnancy loss.

Here, we present population-level estimates of pregnancy loss in rural Bangladesh, reporting both rates and proportions of overall pregnancy loss, miscarriage/spontaneous abortion, and induced abortion separately. These estimates are derived from the Baliakandi HDSS, which includes a dedicated pregnancy surveillance system (PSS) that enables systematic identification, follow-up, and outcome ascertainment for pregnancies [21]. We also report the timing of pregnancy loss and explore the factors associated with its occurrence.

## METHODS

### Study design and setting

We used data from the ongoing HDSS of the International Centre for Diarrhoeal Disease Research, Bangladesh (icddr,b), located in the rural Baliakandi sub-district of Rajbari district [21]. Established in 2017 by the Child Health and Mortality Prevention Surveillance (CHAMPS) network, the Baliakandi HDSS is a routine surveillance system that identifies households using Geographic Information System mapping. It covers a population of over 216 000 people within an area of approximately 239 km^2^ and maintains regularly updated data on births, deaths, marriages, divorces, migration, and sociodemographic characteristics, collected through two-monthly household visits.

The Baliakandi HDSS identifies and follows pregnancies through a PSS. During the routine two-monthly visit, data collectors ask women about the date of their last menstrual period (LMP) and offer a pregnancy test if a married woman of reproductive age has missed her period or cannot recall when it occurred. Women who self-report as pregnant are asked to recall the day on which their LMP began, and, where available, data collectors verify the reported LMP date using ultrasound results. If a woman does not have an ultrasound report, the HDSS offers a pregnancy test to confirm her pregnancy status. The pregnancy surveillance system follows up on each pregnancy to its outcomes, *i.e.* pregnancy loss, stillbirth, or live birth. When pregnancy loss is identified, the data collector asks follow-up questions to the women regarding intention (spontaneous *vs*. induced) and procedure (medical *vs*. non-medical) for classification.

### Study population

We included two groups of participants to determine the burden of pregnancy loss in the surveillance system: women of reproductive age (15–49 years) who were ever married on 1 August 2021 (whom we followed up until 30 June 2023) and women who had a confirmed pregnancy with an LMP between 1 August 2021 and 30 November 2022 to ensure that each woman was observed for at least 28 weeks to document her pregnancy outcome (Figure S1 in the **Online Supplementary Document**).

### Statistical analysis

The primary outcome of interest was pregnancy loss, operationalised as the death of a foetus before 28 weeks of gestation due to miscarriage/spontaneous abortion, or induced abortion [16]. Miscarriage/spontaneous abortion was defined as the loss of a pregnancy before the completion of 28 weeks, unintentionally or through the side effect of medication to maintain the pregnancy, due to other illness, or any trauma or accident. However, if the pregnancy was ended by medical or non-medical means and the length of the pregnancy was shorter than 28 weeks, we categorised it as an induced abortion, as defined elsewhere [22]. We categorised induced abortion as medical or non-medical. Specifically, an induced abortion was considered ‘medical’ if a pregnancy was terminated before 28 weeks through any medical (allopathic) procedure. If a pregnancy was intentionally terminated using any form of medical treatment outside of allopathic medicine (*e.g. *homoeopathic, Ayurvedic, Unani, Kabiraj, or Ojha medicine, water immersion, herbal remedies, *etc*.), we considered it ‘non-medical’. To reduce misclassification between miscarriage/spontaneous and induced abortions in this sensitive legal and social context, data collectors used a structured decision algorithm based on a standardised set of probing questions about the circumstances of each pregnancy loss. Women were asked, in sequence, whether they had wished to continue the pregnancy, whether they had taken any medicines, injections, surgical procedures, or traditional/home remedies ‘to bring on bleeding’ or ‘to remove the pregnancy’, and which type of provider and facility (if any) was involved. Using predefined rules, interviewers then classified each event as medical induced abortion, non-medical induced abortion, or miscarriage/spontaneous, rather than relying on a single self-reported label. Events in which women reported no intention to terminate the pregnancy, but mentioned taking medicines for other illnesses or to maintain the pregnancy, or receiving treatment for complications, were classified as spontaneous abortions unless a method was explicitly described as being used with the primary intention to end the pregnancy.

We identified several covariates based on a literature search and matched them with the variables available in the HDSS data set [22–24]. The explanatory variables were the age (≤19, 20–24, 25–29, 30–34, ≥35 years), education (no education, primary incomplete, primary complete, secondary incomplete, secondary complete or higher), and profession (not involved in income-generating activity, involved in income-generating activity) of both the women and their husbands, education of the women’s mother (no education, primary incomplete, primary complete, secondary incomplete, secondary complete or higher), wealth quintile of the household (based on the wealth index score of the Demographic and Health Survey [25]: lowest, second, middle, fourth, highest), family size (≤4, ≥5), number of children (0, 1, 2, ≥3), history of pregnancy loss (no, yes), history of stillbirth (no, yes), union (Baharpur, Baliakandi, Islampur, Jamalpur, Jangal, Narua, Nawabpur), and marital status of women (married, divorce/separated, widowed).

We reported the respondents’ characteristics first using frequencies and percentages for the two groups. Then, we calculated the rate and proportion of miscarriage/spontaneous abortion and induced abortion, presenting them alongside 95% confidence intervals (CIs). We graphically presented the distribution of miscarriages/spontaneous abortion and induced abortion across gestational weeks, together with different types of pregnancy loss among women.

Given the hierarchical structure of the HDSS data (pregnancy events nested within women, nested within *bari*), we applied a generalised linear mixed model (GLMM) with a logit link to identify associated factors for pregnancy loss. There were 7612 pregnancy events recorded among 7397 women residing in 525 *baris* nested within 276 villages (mean 1.03 pregnancy events per woman and 14.5 events per *bari*). This structure, with pregnancy events clustered within women, women within *baris*, and *baris* within villages, suggested that a multilevel modelling approach was appropriate, which was confirmed by an intra-cluster correlation coefficient of 29.19% (95% CI = 13.8–51.5) [26]. We included random intercepts for both village and *bari* to account for this clustering. We classified pregnancy loss as ‘0’ if it did not occur and ‘1’ if it did. We did not include women who were lost to follow-up and had no pregnancy loss. Furthermore, as a foetus lost beyond the 28th week of gestation is regarded as a stillbirth in Bangladesh, we did not consider it as pregnancy loss.

We selected candidate variables for the multivariable models from the bivariate analysis using a liberal screening threshold (*P* < 0.20), complemented by *a priori* inclusion of covariates with established biological or contextual relevance. We assessed multicollinearity among predictors using variance inflation factors and obtained a mean VIF of 2.8, which indicated no problematic collinearity. Missing data were handled using complete-case analysis. We reported adjusted odds ratios (aORs) with 95% CIs and fitted two separate GLMMs for miscarriage/spontaneous abortion and induced abortion to allow associated factors for these distinct outcomes to be estimated independently.

We used Stata, version 17.2 (StataCorp, College Station, Texas, USA) for data analysis. Associations were considered statistically significant at *P* < 0.05.

## RESULTS

We included 61 428 married women aged 15–49 with 103 965 person-years of observation and 7612 pregnancies. Just above a third (36%) of the married women were aged ≥35 years or older, while just under a third (30%) of pregnant women were aged 20–24 years. About half (51%) and slightly above a third (36%) of married and pregnant women husbands’ aged ≥35 years or older, respectively. Approximately 41% of married women and 52% of pregnant women had incomplete secondary education. More than 90% of women in both groups were not engaged in income-generating activities, while most husbands were economically active. Around 55% women in both samples lived in households with ≤4 members. About 18% of married women and 19% of pregnant women reported a history of pregnancy loss, while only 4% in each group reported a history of stillbirth (**Table 1**).

**Table 1:** Distribution of married women and pregnant women by sociodemographic characteristics, n (%)

	Married women (n = 61 428)*	Pregnant women (n = 7612)†
**Total**		7612 (100)
**Age of women in years**		
15–19	11 258 (18.3)	2286 (30.0)
20–24	9745 (15.9)	2309 (30.3)
25–29	8990 (14.6)	1658 (21.8)
30–34	9263 (15.1)	972 (12.8)
≥35	22 172 (36.1)	387 (5.1)
**Age of husbands in years**		
15–19	1589 (2.6)	371 (4.9)
20–24	5142 (8.4)	1256 (16.5)
25–29	7228 (11.8)	1512 (19.9)
30–34	8475 (13.8)	1736 (22.8)
≥35	31 755 (51.7)	2737 (36.0)
Missing	7239 (11.8)	719 (9.5)
**Education of the women**		
No education	8919 (14.5)	194 (2.6)
Primary incomplete	7437 (12.1)	532 (7.0)
Primary complete	5902 (9.6)	537 (7.1)
Secondary incomplete	25 373 (41.3)	3971 (52.2)
Secondary complete or higher	13 797 (22.5)	2378 (31.2)
**Education of the husband**		
No education	14 582 (23.7)	864 (11.4)
Primary incomplete	9498 (15.5)	1343 (17.6)
Primary complete	6502 (10.6)	915 (12.0)
Secondary incomplete	12 691 (20.7)	1874 (24.6)
Secondary complete or higher	10 887 (17.7)	1894 (24.9)
Missing	7268 (11.8)	722 (9.5)
**Education of the women’s mothers**		
No education	3930 (6.4)	782 (10.3)
Primary incomplete	1418 (2.3)	353 (4.6)
Primary complete	934 (1.5)	242 (3.2)
Secondary incomplete	1367 (2.2)	331 (4.4)
Secondary complete or higher	184 (0.3)	25 (0.3)
Missing	53 595 (87.3)	5879 (77.2)
**Profession of women**		
Not involved in income-generating activities	56 712 (92.3)	7257 (95.3)
Involved in income-generating activities	4716 (7.7)	355 (4.7)
**Profession of husbands**		
Not involved in income-generating activities	2739 (4.5)	551 (7.2)
Involved in income-generating activities	51 450 (83.8)	6342 (83.3)
Missing	7239 (11.8)	719 (9.5)
**Wealth quintile**		
Lowest	11 340 (18.5)	1289 (16.9)
Second	11 700 (19.1)	1419 (18.6)
Middle	12 375 (20.2)	1572 (20.7)
Fourth	13 285 (21.6)	1748 (23.0)
Highest	12 697 (20.7)	1584 (20.8)
Missing	31 (0.1)	-
**Family size (number)**		
≤4	33 662 (54.8)	4185 (55.0)
≥5	27 766 (45.2)	3427 (45.0)
**Number of children (Live Birth)**		
0	5896 (9.6)	703 (9.2)
1	15 143 (24.7)	2734 (35.9)
2	19 617 (32.9)	2439 (32.0)
≥3	20 038 (32.7)	1732 (22.8)
Missing	734 (1.2)	4 (0.1)
**History of pregnancy loss**		
No	50 196 (81.7)	6164 (81.0)
Yes	11 232 (18.3)	1448 (19.0)
**History of stillbirth**		
No	59 000 (96.1)	7336 (96.4)
Yes	2428 (4.0)	276 (3.6)
**Union**		
Baharpur	9672 (15.8)	1280 (16.8)
Baliakandi	7884 (12.8)	1051 (13.8)
Islampur	8667 (14.1)	1178 (15.5)
Jamalpur	8471 (13.8)	1146 (15.1)
Jangal	4683 (7.6)	481 (6.3)
Narua	7005 (11.4)	977 (12.8)
Nawabpur	11 010 (17.9)	1499 (19.7)
Missing	4036 (12.7)	-
**Current marital status of women**		
Married	59 061 (96.2)	7606 (99.9)
Widowed	1328 (2.2)	0 (0.0)
Divorce/separated	1039 (1.7)	6 (0.1)

*Women with reproductive age (15–49 years) on 1 August 2021.

†Last menstrual period between 1 August 2021 to 30 November 2022.

**Figure 1:**
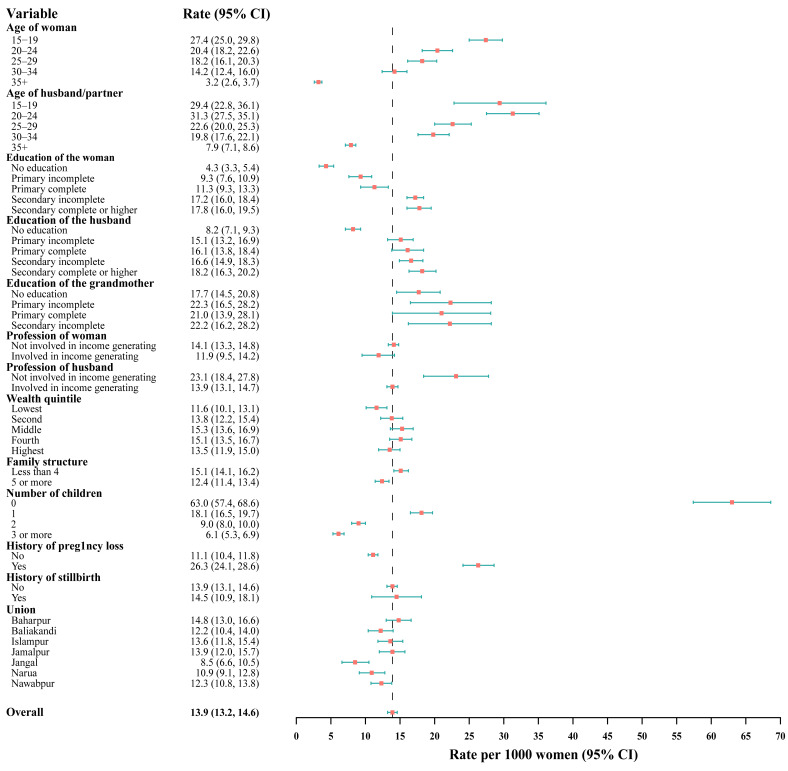
Rate of pregnancy loss per 1000 women per year among married women.

The overall rate of pregnancy loss per 1000 married women per year was 13.9 (95% CI = 13.2–14.6). The rates of miscarriage/spontaneous abortions and induced abortions per 1000 ever-married women per year were 10.6 (95% CI = 10.0–11.2) and 3.3 (95% CI = 3.0–3.7), respectively (Table S1 in the **Online Supplementary Document**). The rate of pregnancy loss declined with increasing age and parity, being highest among women with no children (63.0 per 1000) and lowest among those with three or more children (6.1 per 1000). In contrast, pregnancy loss increased with women’s education level (*e.g.* no education: 4.3 per 1000 *vs*. secondary complete or higher: 17.8 per 1000). It was also significantly higher in smaller households (≤4 members: 15.1 per 1000 *vs*. ≥5 members: 12.4 per 1000). Women not engaged in income-generating activities had higher loss rates than those who were (14.1 *vs*. 11.9 per 1000), and a similar pattern was observed for husbands’ employment (not involved: 23.1 *vs*. involved: 13.9 per 1000). Pregnancy loss was also higher among women with a previous history of pregnancy loss (26.3 *vs*. 11.1 per 1000) (**Figure 1**).

**Figure 2:**
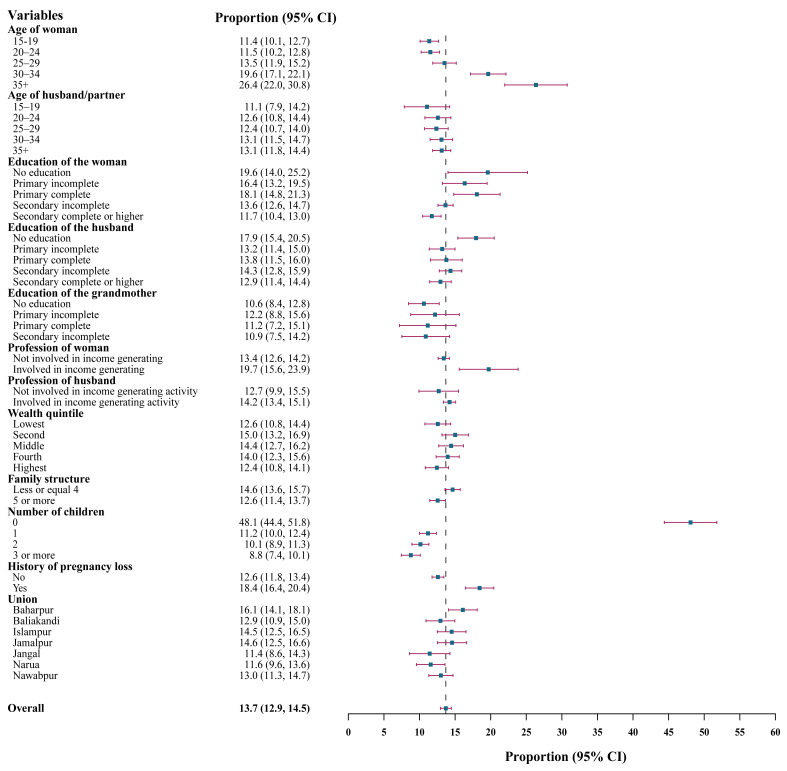
Proportion (%) of pregnancy loss among pregnant women.

The overall proportion of pregnancy loss among pregnant women was 14% (95% CI = 13–15). The proportions of miscarriage/spontaneous abortions and induced abortions among pregnant women were 11% and 3%, respectively (Table S2 in the **Online Supplementary Document**). As the age of pregnant women increased, the proportion of pregnancy loss also rose. We noted a similar increasing pattern across higher levels of women’s education. Women engaged in income-generating activities experienced a higher proportion of pregnancy loss (20%) than those not working for income (13%). In contrast, family size and number of children showed an inverse relationship with pregnancy loss. Pregnant women with a previous history of pregnancy loss again had a higher proportion of loss in the current pregnancy (18% *vs*. 13%) (**Figure 2**).

The rates of miscarriage/spontaneous abortion and induced abortion were 10.6 and 3.3 per 1000 ever-married women-years, respectively (Table S2 in the **Online Supplementary Document**). Miscarriage/spontaneous abortion rates decreased with women’s age, whereas induced abortion rates tended to increase with husbands’ age. For both outcomes, rates increased with higher education for women and husbands and were higher when either partner was not engaged in income-generating work. Miscarriage/spontaneous abortion and induced abortion were also more frequent among women with smaller families, fewer children, prior pregnancy loss, and prior induced abortion. Women with a history of stillbirth had a slightly higher rate of induced abortion (4.5 *vs*. 3.3 per 1000 ever-married women‑years)

About 11% of currently pregnant women experienced miscarriage/spontaneous abortion, while 3% experienced induced abortion (Table S3 in the **Online Supplementary Document**). The proportion of both outcomes rose with women’s age but showed the opposite pattern with husbands’ age. Miscarriages/spontaneous abortions were more common among women in income-generating work and those with smaller families and fewer children, whereas induced abortions were relatively more frequent in poorer households and larger families. A history of pregnancy loss or stillbirth was associated with higher proportions of both miscarriages/spontaneous abortions and induced abortions.

In terms of women’s age, advanced age was significantly associated with higher odds of spontaneous abortion. Odds for induced abortion were significantly higher among women aged 30–34 years (aOR = 4.78; 95% CI = 1.99–11.44) and ≥35 years (aOR = 12.73; 95% CI = 4.12–39.34). Higher husbands’ age was also associated with increased odds of miscarriage/spontaneous abortion. Women with secondary or higher education had lower odds of induced abortion (aOR = 0.22; 95% CI = 0.09–0.55), and husbands with secondary or higher education had lower odds of spontaneous abortion (aOR = 0.64; 95% CI = 0.43–0.96). Regarding occupation, pregnant women whose husbands were engaged in income-generating activities had higher odds of miscarriage/spontaneous abortion (aOR = 1.63; 95% CI = 1.08–2.48). The number of children showed a protective association with spontaneous abortion, with the odds decreasing as parity increased. Finally, a history of pregnancy loss or stillbirth was associated with higher odds of miscarriage/spontaneous abortion (aOR = 1.34; 95% CI = 1.04–1.72), while it was not significantly associated with induced abortion (**Table 2**).

**Table 2:** Proportion and determinants of miscarriage/spontaneous abortion and induced abortion among pregnant women

	Miscarriage/spontaneous abortion					Induced abortion				
	**% (95% CI)**	**OR (95% CI)**	***P*-value**	**aOR (95% CI)**	***P*-value**	**% (95% CI)**	**OR (95% CI)**	***P*-value**	**aOR (95% CI)**	***P*-value**
**Age of women in years**										
15–19	9.5 (8.3–10.7)	ref		ref		1.8 (1.3–2.4)	ref		ref	
20–24	9.3 (8.1–10.5)	0.97 (0.80–1.19)	0.794	3.60 (2.46–5.28)	<0.001	2.2 (1.6–2.8)	1.21 (0.80–1.82)	0.372	1.29 (0.72–2.31)	0.397
25–29	11.0 (9.5–12.5)	1.17 (0.95–1.44)	0.139	11.03 (6.45–18.86)	<0.001	2.5 (1.8–3.3)	1.39 (0.90–2.14)	0.137	1.63 (0.81–3.28)	0.174
30–34	13.6 (11.4–15.7)	1.49 (1.18–1.88)	0.001	25.83 (13.10–50.92)	<0.001	6.1 (4.6–7.6)	3.45 (2.31–5.17)	<0.001	4.78 (1.99–11.44)	<0.001
≥35	14.5 (11.0–18.0)	1.6 (1.17–2.20)	0.003	38.94 (17.21–88.14)	<0.001	11.9 (8.7–15.1)	7.21 (4.67–11.12)	<0.001	12.73 (4.12–39.34)	<0.001
**Age of husband in years**										
15–19	8.4 (5.5–11.2)	ref		ref		2.7 (1.1–4.3)	ref		ref	
20–24	11.0 (9.3–12.7)	1.35 (0.90–2.04)	0.146	1.85 (1.05–3.28)	0.029	1.6 (0.9–2.3)	0.58 (0.27–1.26)	0.17	0.63 (0.26–1.54)	0.309
25–29	10.6 (9.1–12.2)	1.31 (0.87–1.95)	0.192	2.31 (1.27–4.21)	<0.001	1.7 (1.1–2.4)	0.63 (0.30–1.32)	0.223	0.68 (0.27–1.69)	0.407
30–34	9.6 (8.2–11.0)	1.17 (0.78–1.74)	0.449	2.79 (1.49–5.24)	<0.001	3.5 (2.6–4.3)	1.29 (0.66–2.55)	0.459	1.24 (0.50–3.06)	0.642
≥35	9.2 (8.1–10.3)	1.56 (1.05–2.30)	0.026	3.69 (1.90–7.16)	<0.001	3.9 (3.2–4.7)	2.04 (1.06–3.94)	0.033	0.94 (0.36–2.46)	0.892
**Education of the women**										
No education	9.3 (5.2–13.4)	ref		ref		10.3 (6.0–14.6)	ref		ref	
Primary incomplete	12.0 (9.3–14.8)	1.34 (0.77–2.32)	0.301	2.27 (1.08–4.74)	0.030	4.3 (2.6–6.1)	0.39 (0.21–0.73)	0.003	0.52 (0.22–1.21)	0.128
Primary complete	11.4 (8.7–14)	1.25 (0.72–2.18)	0.424	1.98 (0.95–4.15)	0.070	6.7 (4.6–8.8)	0.63 (0.35–1.11)	0.108	1.09 (0.50–2.39)	0.829
Secondary incomplete	10.5 (9.5–11.4)	1.14 (0.70–1.88)	0.594	1.64 (0.83–3.22)	0.153	3.2 (2.6–3.7)	0.29 (0.17–0.47)	<0.001	0.57 (0.27–1.18)	0.129
Secondary complete or higher	10.3 (9.0–11.5)	1.12 (0.68–1.85)	0.664	0.76 (0.37–1.55)	0.446	1.5 (1.0–2.0)	0.13 (0.07–0.23)	<0.001	0.22 (0.09–0.55)	0.001
**Education of the husbands**										
No education	11.7 (9.5–13.8)	ref		ref		6.3 (4.6–7.9)	ref		ref	
Primary incomplete	9.4 (7.8–10.9)	0.78 (0.59–1.03)	0.082	0.79 (0.54–1.15)	0.212	3.8 (2.8–4.8)	0.59 (0.40–0.88)	0.009	0.86 (0.52–1.43)	0.569
Primary complete	11.0 (9.0–13.1)	0.94 (0.70–1.26)	0.665	0.96 (0.65–1.43)	0.848	2.7 (1.7–3.8)	0.42 (0.26–0.68)	<0.001	0.55 (0.30–1.02)	0.057
Secondary incomplete	11.2 (9.7–12.6)	0.95 (0.74–1.22)	0.68	0.81 (0.56–1.16)	0.248	3.2 (2.4–4.0)	0.50 (0.34–0.72)	<0.001	0.78 (0.47–1.29)	0.333
Secondary complete or higher	11.1 (9.7–12.6)	0.95 (0.74–1.22)	0.673	0.64 (0.43–0.96)	0.032	1.8 (1.2–2.4)	0.27 (0.18–0.42)	<0.001	0.61 (0.33–1.14)	0.125
**Profession of women**										
Not involved in income-generating activities	10.4 (9.7–11.1)	ref		ref		3 (2.7–3.4)	ref		ref	
Involved in income-generating activities	14.4 (10.7–18.0)	1.45 (1.07–1.97)	0.017	1.32 (0.84–2.08)	0.227	5.4 (3.0–7.7)	1.80 (1.11–2.91)	0.017	1.13 (0.58–2.21)	0.721
**Profession of husbands**										
Not involved in income-generating activities	11.4 (8.8–14.1)	ref		ref		1.3 (0.3–2.2)	ref		ref	
Involved in income-generating activities	10.8 (10.0–11.6)	0.94 (0.71–1.23)	0.647	1.63 (1.08–2.48)	0.021	3.4 (3.0–3.9)	2.75 (1.29–5.87)	0.009	1.64 (0.68–3.98)	0.275
**Wealth quintile**										
Lowest	9.3 (7.7–10.9)	ref		ref		3.3 (2.3–4.2)	ref		ref	
Second	10.8 (9.2–12.4)	1.18 (0.92–1.51)	0.204	1.22 (0.86–1.72)	0.259	4.2 (3.2–5.3)	1.31 (0.88–1.96)	0.187	1.52 (0.91–2.53)	0.110
Middle	11.5 (9.9–13.0)	1.26 (0.99–1.61)	0.063	1.15 (0.82–1.62)	0.414	3 (2.2–3.8)	0.92 (0.60–1.40)	0.681	1.12 (0.66–1.89)	0.670
Fourth	11.1 (9.6–12.6)	1.22 (0.96–1.55)	0.11	1.06 (0.76–1.49)	0.738	2.9 (2.1–3.7)	0.87 (0.58–1.33)	0.527	1.02 (0.60–1.74)	0.937
Highest	9.8 (8.4–11.3)	1.06 (0.83–1.37)	0.626	0.71 (0.48–1.04)	0.079	2.6 (1.8–3.4)	0.79 (0.51–1.22)	0.287	1.37 (0.75–2.48)	0.305
**Family size**										
≤4	11.8 (10.8–12.8)	ref		ref		2.8 (2.3–3.4)	ref		ref	
≥5	9.0 (8.1–10.0)	0.74 (0.64–0.86)	<0.001	0.91 (0.74–1.13)	0.412	3.5 (2.9–4.2)	1.25 (0.97–1.62)	0.088	1.67 (1.18–2.35)	0.004
**Number of children**										
0	45.9 (42.3–49.6)	ref		ref		2.1 (1.1–3.2)	ref		ref	
1	9.1 (8.0–10.1)	0.12 (0.10–0.14)	<0.001	0.04 (0.02–0.06)	<0.001	2.1 (1.6–2.7)	0.99 (0.56–1.76)	0.984	0.82 (0.40–1.67)	0.586
2	6.5 (5.5–7.5)	0.08 (0.07–0.10)	<0.001	0.01 (0.00–0.01)	<0.001	3.6 (2.9–4.4)	1.74 (1.00–3.02)	0.051	0.83 (0.39–1.76)	0.627
≥3	4.3 (3.3–5.2)	0.05 (0.04–0.07)	<0.001	0.0008 (0.0003–0.0025)	<0.001	4.5 (3.5–5.5)	2.16 (1.24–3.79)	0.007	0.32 (0.13–0.80)	0.015
**History of pregnancy loss/stillbirth**										
No	9.9 (9.1–10.6)	ref		ref		2.7 (2.3–3.1)	ref		ref	
Yes	13.3 (11.6–15.0)	1.27 (1.07–1.51)	0.006	1.34 (1.04–1.72)	0.024	5.1 (4.0–6.2)	1.87 (1.41–2.48)	<0.001	1.23 (0.85–1.77)	0.274
**Union**										
Baharpur	13 (11.1–14.8)	ref		ref		3.1 (2.2–4.1)	ref		ref	
Baliakandi	9.9 (8.1–11.7)	0.74 (0.57–0.96)	0.021	0.86 (0.60–1.25)	0.433	3.0 (2.0–4.1)	0.97 (0.61–1.56)	0.911	0.96 (0.54–1.73)	0.901
Islampur	10.4 (8.6–12.1)	0.78 (0.6–0.99)	0.045	0.62 (0.43–0.89)	0.010	4.2 (3.0–5.3)	1.35 (0.88–2.06)	0.172	1.24 (0.72–2.12)	0.436
Jamalpur	10.2 (8.5–12.0)	0.76 (0.59–0.98)	0.035	0.83 (0.58–1.18)	0.297	4.4 (3.2–5.6)	1.41 (0.93–2.16)	0.109	1.34 (0.79–2.30)	0.281
Jangal	7.9 (5.5–10.3)	0.58 (0.4–0.83)	0.003	0.53 (0.31–0.89)	0.016	3.5 (1.9–5.2)	1.14 (0.64–2.02)	0.666	1.08 (0.53–2.23)	0.832
Narua	9.9 (8.1–11.8)	0.74 (0.57–0.96)	0.026	0.70 (0.48–1.02)	0.060	1.6 (0.8–2.4)	0.52 (0.29–0.93)	0.027	0.50 (0.25–1.00)	0.050
Nawabpur	10.6 (9.0–12.2)	0.80 (0.63–1.00)	0.054	0.73 (0.52–1.02)	0.063	2.4 (1.6–3.2)	0.76 (0.48–1.20)	0.245	0.76 (0.43–1.34)	0.343

aOR – adjusted odds ratio, CI – confidence interval, ref – reference

**Figure 3:**
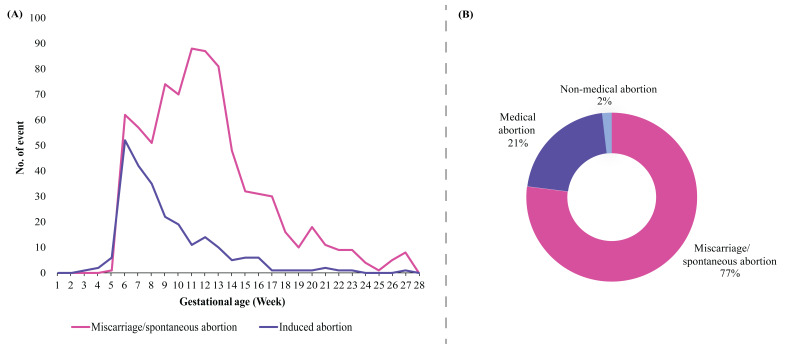
Timing (**Panel A**) and types (**Panel B**) of pregnancy loss among pregnant women.

In terms of the timing of pregnancy loss, events of miscarriage/spontaneous abortion and induced abortion remained relatively low in the early weeks of gestation and progressively increased from week 5. Furthermore, the incidence of induced abortions peaked around the 6th to 7th weeks of gestation. In contrast, miscarriages/spontaneous abortions peaked the 11th to 13th weeks of gestation. In terms of types of pregnancy loss, the majority (77%) of pregnant women had miscarriages/spontaneous abortions, followed by medical (21%) and non-medical abortions (2%) (**Figure 3**).

## DISCUSSION

Pregnancy loss has significant physical, psychological, and social consequences for women and remains an important, but under-recognised public health issue [27]. We found that approximately 14 per 1000 married women in Bangladesh experienced pregnancy loss annually, and that 14% of recognised pregnancies resulted in pregnancy loss. Comparisons of pregnancy loss across countries and studies should be interpreted with caution, as operational definitions of pregnancy loss, including gestational age thresholds and inclusion of miscarriage, stillbirth, or induced abortion, differ across settings. In our sample, higher maternal and paternal age and a history of prior pregnancy loss/stillbirth were associated with miscarriage, while higher maternal age and larger family size were associated with induced abortion.

The proportion of pregnancy loss in our study was lower than that reported in Nepal (20%) [28], but higher than in India (11%) [29]. The estimate of pregnancy loss in Nepal is derived from a nationally representative survey that capture both miscarriage and stillbirth [28], whereas the estimate for India is based on population-based surveys that define pregnancy loss as occurring before 20 weeks of gestation [29]. Our study, based on prospective HDSS surveillance, excluded stillbirths and defined pregnancy loss as foetal loss before 28 weeks of gestation, further limiting direct comparability across settings. Our estimate was also higher than those reported in a systematic review from sub-Saharan Africa (Malawi, South Africa, Uganda, and Zimbabwe), partly because induced abortion was not included in their definition of pregnancy loss [30].

Approximately 1 in 10 pregnancies in our study ended in miscarriage/spontaneous abortion, which is consistent with estimates reported by Das *et al*. [16] and Alam *et al*. [31]. Despite Das *et al*. [16] conducted their study in a coastal area of Bangladesh, while our study enrolled women from a more central region, the similarity in estimates suggests that miscarriage prevalence may be comparable across geographic settings. Our results also align with findings from Alam *et al*. [31], a hospital-based study among ever-married, currently pregnant women, supporting the robustness of miscarriage estimates across different study populations and designs. A 40-year prospective cohort study in Denmark found that around 10% of pregnancies resulted in spontaneous/missed abortion, similar to our findings, despite differences in study design [32]. The global pooled prevalence of miscarriage, estimated at approximately 15%, exceeds our estimate, likely due to differences in population characteristics and study design, as most participants in our study were younger, healthier community-dwelling women with lower risks associated with chromosomal abnormalities, advanced maternal age, and comorbidities [2]. Compared with other South Asian countries, our estimate was higher than in India and Nepal, highlighting the importance of region- and population-specific data [29,33].

About 3% of recognised pregnancies in our study ended in induced abortion, an estimate similar to the 4% reported by Das *et al*. [16]. However, our estimate differed substantially from that of Singh *et al*. [17], who reported around 18 induced abortions per 1000 women aged 15–44 years. This variance in the estimate of induced abortion can be attributed to several reasons, including differences in research design and methodology. Singh *et al*. [34] employed an indirect approach, which involved a health facility survey, interviews with key informants, and data on menstrual regulation and post-abortion care handled at facilities, to estimate the rate of induced abortions. In contrast, we adopted a more direct approach by utilising data from an HDSS site visited every two months by trained data collectors. This reduced the possibility of inadequate pregnancy outcome reports or of pregnant women withholding information from data collectors. Although there may be some misreporting of induced abortions as miscarriages due to stigma, this would lead to an overall reduced estimate of induced abortion.

Our findings indicate that miscarriage/spontaneous abortion is significantly associated with maternal age advancement. A Norwegian study found that women in their late twenties have the lowest odds of miscarriage, which increases substantially in women aged >45 years [35]. Another study found that high maternal age was significantly associated with miscarriage/spontaneous abortion, independent of parity, calendar period, or history of miscarriages [36]. Pregnancy loss is more prevalent among women in their late 30s or older, regardless of reproductive history; hence, it is advised that this be taken into consideration while planning a pregnancy and receiving prenatal counselling [36]. Women must obtain counselling and prenatal care to evaluate any potential hazards and address any difficulties that may arise during pregnancy.

On the contrary, the risk of an induced abortion was considerably greater for women aged 30–34. This finding was consistent with another study in Bangladesh, which found that the prevalence of menstrual regulation rose with women's age, peaking in the older age range of ≥30 years [37]. The aforementioned study also observed higher odds of miscarriage/spontaneous abortion with increasing husband age. Previous research has consistently reported an association between advanced paternal age and miscarriage [38,39]. Evidence from a systematic review and meta-analysis shows that advanced paternal age increases miscarriage risk primarily due to accumulating genetic and epigenetic defects in sperm, including higher rates of de novo mutations, chromosomal aneuploidy, and imprinting errors, which impair embryo development and implantation [38].

Pregnant women who had previously experienced pregnancy loss or stillbirth in our study were more likely to have a miscarriage/spontaneous abortion, which is consistent with prior research [40–43]. Similarly, an earlier systematic review showed that the risk of miscarriage was lowest in women without a history of miscarriage, increasing by around 10% for each subsequent loss and reaching 42% in women with ≥3 prior miscarriages [2]. We could not obtain information on the number of prior pregnancy losses in the HDSS database. Therefore, we could not examine any possible dose-response association between previous and future pregnancy loss. Among the biological causes of miscarriages/spontaneous abortions are chromosomal abnormalities, uterine abnormalities, infections, immunological issues, and hormone imbalances [44,45]. This finding may also be influenced by some women using menstrual regulation as a method of family planning. In our study, women with a history of pregnancy loss had incidence rates of 20 miscarriages/spontaneous abortions and 6 induced abortions per 1000 women per year. The association between prior pregnancy loss or stillbirth and induced abortion was not statistically significant. Due to a lack of data, we could not distinguish whether previous losses were miscarriages or induced abortions.

Women who belonged to larger families had significantly higher odds of induced abortion. While there is a scarcity of literature on this relationship, some studies have suggested that the link between larger family size and abortion can be influenced by a range of factors, such as access to reproductive health services, restrictive state policies, family planning outcomes, health care system factors, provider-related issues, and socioeconomic determinants, *etc*. [46-48]. We also examined the relationship between family size and the odds of induced abortions and found that, even after adjusting for wealth quintiles, a larger family was associated with higher odds of induced abortion in the final model. This finding indicates that mothers are not only concerned about the economic burden, but also about their child's future and the practicalities of managing an additional family member.

Regarding women’s education, we found that women who had completed secondary or higher education had lower odds of induced abortion compared to women with lower levels of education. Another study in Russia further confirmed the protective effect of higher education on induced abortion probability [49]. As women's education increases, their awareness of the danger signs that may occur during pregnancy will increase, and they will have a better lifestyle overall [50,51].

The incidence of both spontaneous and induced abortion in our study progressively increased from week 5 of gestation, with most events occurring in the first trimester of pregnancy. Another study in Bangladesh arrived at similar results regarding miscarriage/spontaneous abortion, with miscarriages/spontaneous abortions peaking between 8 and 14 weeks of gestation [16]. In Bangladesh, abortion is illegal except when necessary to save a woman's life [52]. Menstrual regulation, however, has been legal since 1979 and is reasonably accessible to women [53], while menstrual regulation with medication has also been approved for up to 10 weeks since 2012 [52]. Therefore, any termination of pregnancy beyond 10 weeks is legally restricted, which may partly explain our finding that induced abortions were predominantly reported in the six to nine weeks. However, the legal and sociocultural context surrounding abortion in Bangladesh is highly sensitive, with stigma, fear of legal repercussions, and social judgment possibly leading women to conceal induced abortions or report them as miscarriage/spontaneous abortions. In addition, provider reporting practices and women’s self-reporting may favour the use of socially acceptable terminology such as ‘menstrual regulation’, particularly for terminations occurring later in gestation. Consequently, induced abortions occurring beyond the 10 weeks may be underreported or misclassified as miscarriages, potentially inflating estimates of miscarriage/spontaneous abortion. Due to the sensitive nature of the topic and associated legal complexities, the data collectors do not pursue follow-up questions and classify all medically induced abortions as either MR or procedures undertaken to preserve the woman's life. However, a small percentage of abortions are conducted through non-medical methods; such practices continue to occur despite being illegal in Bangladesh [17,54,55].

Lastly, when interpreting our findings, it is important to distinguish between crude descriptive patterns and adjusted associations from the multivariable models. The descriptive tables present unadjusted proportions, which are strongly influenced by underlying differences in group composition [56]. Apparent inconsistencies between descriptive and adjusted results, therefore mostly reflect confounding and clustering of related factors, rather than true contradictions [56].

### Strengths and limitations

The major strengths of this study are the rates and proportions that we derived from two population-level estimates (*i.e*. married women and pregnant women) to explain the total burden of pregnancy loss. Furthermore, this is the first study in Bangladesh to provide a comprehensive estimate of the burden of pregnancy loss, including miscarriage/spontaneous and induced abortions. We collected data from the Baliakandi HDSS, one of the surveillance systems of icddr,b, where a pregnancy surveillance system is run by skilled data collectors through two-monthly rounds. Compared to cross-sectional surveys of a comparable sort, the prospective nature of HDSS data collection ensures the accuracy of LMP dates to a sufficient degree. Therefore, there is less chance of misreporting and recall bias, and error, which improves the overall validity of pregnancy loss reporting.

Yet our study also has some limitations. The estimated burden of pregnancy loss is likely underestimated, as a sizable proportion of women, particularly in low-resource settings, may not have known they were pregnant at the time of the loss, and very early biochemical or pre-clinical pregnancies occurring between the HDSS three-monthly visits would not have been captured. This under-ascertainment is expected to bias our estimates downward compared with studies using prospective pregnancy testing or shorter-interval follow-up. Furthermore, misclassification of pregnancy loss is possible: we classified induced abortions as ‘medical’ or ‘non-medical’ solely on the basis of self-report, in a highly sensitive and stigmatised context where women may be reluctant to disclose abortion. In the absence of triangulation with facility records, community validation, or qualitative verification, there is a substantial risk of systematic under-reporting and misclassification of induced abortions, which would further bias estimates toward lower rates. In addition, our estimates come from a single HDSS site and may not represent all pregnant women in Bangladesh. Furthermore, we defined pregnancy loss as foetal death before 28 completed weeks of gestation, consistent with the local convention that losses at or after 28 weeks are classified as stillbirths. This gestational-age threshold may differ from those used in other studies and regions, which affects the comparability of absolute rates and may contribute to discrepancies with regional or global estimates. Finally, the HDSS collects only a limited set of demographic and socioeconomic variables to maintain efficiency, which restricted our ability to examine the influence of more detailed biological, social, and health-system factors on pregnancy loss.

## CONCLUSIONS

A notable burden of pregnancy loss exists in rural Bangladesh, with most events occurring spontaneously and within the initial trimester. Given this scenario, maternal health programmes should implement specific initiatives to engage with pregnant women during the early stages of pregnancy. This proactive approach is essential for effective prevention and protection against pregnancy loss. Additionally, recognising pregnancies with a history of previous loss as high risk is crucial and should prompt closer monitoring to reduce the risk of complications and prevent further losses.

## Additional material


Online Supplementary Document
