# Supplementary material for: Pregnancy loss in rural Bangladesh: an analysis of rates, proportions, timing, and determinants based on data from a Health and Demographic Surveillance System
Source: J Glob Health. 2026 Mar 27;16:04101. doi: 10.7189/jogh.16.04101 (PMC13030114; doi:10.7189/jogh.16.04101)
Supplement: Online Supplementary Document [file jogh-16-04101-s001.pdf]

Supplement to: Raza S, Banik R, Noor STA, Rahman QS, Zahan FN, Siddique AB, Hasan MM, Majid T, Jahan E, Sayeed A, Hossain L, Ether ST, Rahman A, Huq N, Arifeen SE, Ahmed A, Rahman AE. Pregnancy loss in rural Bangladesh: an analysis of rates, proportions, timing, and determinants based on data from a Health and Demographic Surveillance System. J Glob Health. 2026;16:04101.

Table S1. Rate of pregnancy loss, spontaneous abortion, and induced abortion among all study women

**Variables**	**Women (Ever married on August 1st, 2021)**	**Person-year observation**	**Number of pregnancies (LMP on August 1st, 2021)**	**Events observed: miscarriage/ spontaneous abortion**	**Events observed -inducing abortion**	**Medically Terminated Miscarriage**	**Non-Medically Terminated Miscarriage**	**Rate of miscarriage/ spontaneous abortion**	**95% CI**	**Rate of induced abortion**	**95% CI**	**Rate of pregnancy loss**	**95% CI**
	**n**	**n**	**n**	**n**	**n**	**n**	**n**	**Per 1000 women per year**		**Per 1000 women per year**		**Per 1000 women per year**	
**Total (N)**	**61,428**	**103,965**	**10,527**	**1,102**	**343**	**312**	**31**	**10.60**	**(9.98,11.22)**	**3.30**	**(2.95,3.65)**	**13.90**	**(13.19,14.61)**
**Age of woman**													
15-19	11,258	17,470	4,111	387	92	85	7	22.15	(19.97,24.34)	5.27	(4.19,6.34)	27.42	(25,29.84)
20-24	9,745	15,963	2,801	265	60	55	5	16.60	(14.62,18.58)	3.76	(2.81,4.71)	20.36	(18.17,22.55)
25-29	8,990	15,513	1,988	214	69	60	9	13.80	(11.96,15.63)	4.45	(3.4,5.5)	18.24	(16.14,20.35)
30-34	9,263	16,644	1,181	167	70	63	7	10.03	(8.52,11.55)	4.21	(3.22,5.19)	14.24	(12.44,16.04)
35+	22,172	38,376	446	69	52	49	3	1.80	(1.37,2.22)	1.36	(0.99,1.72)	3.15	(2.59,3.71)
**Age of Husband**													
15-19	1,589	2,482	638	49	24	21	3	19.74	(14.27,25.21)	9.67	(5.82,13.52)	29.41	(22.76,36.05)
20-24	5,142	8,113	1,932	215	39	37	2	26.50	(23.01,30)	4.81	(3.3,6.31)	31.31	(27.52,35.1)
25-29	7,228	12,204	2,372	220	56	50	6	18.03	(15.67,20.39)	4.59	(3.39,5.79)	22.62	(19.98,25.25)
30-34	8,475	14,934	2,264	234	62	56	6	15.67	(13.68,17.66)	4.15	(3.12,5.18)	19.82	(17.59,22.06)
35+	31,755	56,226	2,432	301	141	129	12	5.35	(4.75,5.96)	2.51	(2.09,2.92)	7.86	(7.13,8.59)
missing	7,239	10,006	889	83	21	19	2						
**Education of the woman**													
No education	8,919	14,788	299	34	30	28	2	2.30	(1.53,3.07)	2.03	(1.3,2.75)	4.33	(3.27,5.39)
Primary incomplete	7,437	12,926	770	83	37	35	2	6.42	(5.04,7.8)	2.86	(1.94,3.78)	9.28	(7.63,10.94)
Primary complete	5,902	10,448	743	74	44	43	1	7.08	(5.47,8.69)	4.21	(2.97,5.45)	11.29	(9.27,13.32)
Secondary incomplete	25,373	44,044	5,411	575	181	161	20	13.06	(11.99,14.12)	4.11	(3.51,4.71)	17.16	(15.95,18.38)
Secondary complete or higher	13,797	21,759	3,304	336	51	45	6	15.44	(13.8,17.08)	2.34	(1.7,2.99)	17.79	(16.03,19.54)
missing													
**Education of the husband**													
No education	14,582	25,594	1,239	138	71	65	6	5.39	(4.49,6.29)	2.77	(2.13,3.42)	8.17	(7.06,9.27)
Primary incomplete	9,498	16,804	1,876	177	76	68	8	10.53	(8.99,12.08)	4.52	(3.51,5.54)	15.06	(13.21,16.9)
Primary complete	6,502	11,535	1,338	149	37	37	0	12.92	(10.86,14.98)	3.21	(2.18,4.24)	16.13	(13.83,18.42)
Secondary incomplete	12,691	22,228	2,665	280	89	79	10	12.60	(11.13,14.06)	4.00	(3.17,4.83)	16.60	(14.92,18.28)
Secondary complete or higher	10,887	17,755	2,518	275	49	44	5	15.49	(13.67,17.3)	2.76	(1.99,3.53)	18.25	(16.28,20.22)
missing	7,268	10,049	891	83	21	19	2						

**Education of the maternal grandmother**												
No education	3,930	6,517	1,110	96	19	17	2	14.73	(11.81,17.65)	2.92	(1.61,4.22)	17.65	(14.45,20.84)
Primary incomplete	1,418	2417.1	431	45	9	7	2	18.62	(13.23,24.01)	3.72	(1.3,6.15)	22.34	(16.45,28.23)
Primary complete	934	1571.4	308	30	3	3	0	19.09	(12.33,25.86)	1.91	(-0.25,4.07)	21.00	(13.91,28.09)
Secondary incomplete	1,367	2,296	430	41	10	9	1	17.86	(12.44,23.27)	4.36	(1.66,7.05)	22.21	(16.18,28.24)
Secondary complete or higher	184	287	39	4	0	0	0	13.94	(0.37,27.51)	0.00	(0,0)	13.94	(0.37,27.51)
missing	53,595	90,876	8,209	886	302	276	26						
**Profession of woman**													
Not involved in income-generating activity	56,712	95,875	10,014	1037	312	284	28	10.82	(10.16,11.47)	3.25	(2.89,3.61)	14.07	(13.32,14.82)
Involved in income-generating activity	4,716	8,089	513	65	31	28	3	8.04	(6.09,9.98)	3.83	(2.49,5.18)	11.87	(9.51,14.23)
**Profession of Husband**													
Not involved in income-generating activity	2,739	3,940	593	81	10	10	0	20.56	(16.13,24.99)	2.54	(0.97,4.11)	23.09	(18.4,27.78)
Involved in income-generating activity	51,450	90,019	9,045	938	312	283	29	10.42	(9.76,11.08)	3.47	(3.08,3.85)	13.89	(13.12,14.65)
missing	7,239	10,006	889	83	21	19	2						
**Wealth quintile**													
Lowest	11,340	19,710	1,834	164	65	58	7	8.32	(7.05,9.59)	3.30	(2.5,4.1)	11.62	(10.12,13.11)
Second	11,700	20,110	1,970	200	78	72	6	9.95	(8.57,11.32)	3.88	(3.02,4.74)	13.82	(12.21,15.44)
Middle	12,375	21,029	2,198	256	65	58	7	12.17	(10.69,13.66)	3.09	(2.34,3.84)	15.26	(13.61,16.92)
Fourth	13,285	22,255	2,383	258	78	74	4	11.59	(10.19,13)	3.50	(2.73,4.28)	15.10	(13.5,16.7)
Highest	12,697	20,820	2,140	223	57	50	7	10.71	(9.31,12.11)	2.74	(2.03,3.45)	13.45	(11.88,15.01)
missing	31	40	2	1	0	0	0						
**Family size**													
≤4	33,662	56,212	5,779	678	173	160	13	12.06	(11.16,12.96)	3.08	(2.62,3.54)	15.14	(14.13,16.15)
≥5	27,766	47,752	4,748	424	170	152	18	8.88	(8.04,9.72)	3.56	(3.03,4.09)	12.44	(11.45,13.43)
**Number of children**													
0	5,896	7,129	837	428	21	19	2	60.04	(54.53,65.56)	2.95	(1.69,4.2)	62.99	(57.35,68.63)
1	15,143	25,769	5,327	363	103	95	8	14.09	(12.65,15.53)	4.00	(3.23,4.77)	18.08	(16.46,19.71)
2	19,617	35,215	2,581	207	109	97	12	5.88	(5.08,6.68)	3.10	(2.52,3.68)	8.97	(7.99,9.96)
>=3	20,038	35,222	1,779	104	110	101	9	2.95	(2.39,3.52)	3.12	(2.54,3.71)	6.08	(5.26,6.89)
**History of pregnancy loss**													
No	50,196	84,775	8,365	710	230	204	26	8.38	(7.76,8.99)	2.71	(2.36,3.06)	11.09	(10.38,11.79)
Yes	11,232	19,190	2,162	392	113	108	5	20.43	(18.43,22.43)	5.89	(4.81,6.97)	26.32	(24.05,28.58)
**History of stillbirth**													
No	59,000	99,758	10,156	1060	324	295	29	10.63	(9.99,11.26)	3.25	(2.89,3.6)	13.87	(13.15,14.6)
Yes	2,428	4,207	371	42	19	17	2	9.98	(6.98,12.99)	4.52	(2.49,6.54)	14.50	(10.89,18.11)
**Union**													
Baharpur	9,672	17,534	1,642	193	66	61	5	11.01	(9.46,12.55)	3.76	(2.86,4.67)	14.77	(12.99,16.56)
Baliakandi	7,884	14,296	1,398	134	41	36	5	9.37	(7.79,10.95)	2.87	(1.99,3.74)	12.24	(10.44,14.04)
Islampur	8,667	15,734	1,480	151	63	58	5	9.60	(8.07,11.12)	4.00	(3.02,4.99)	13.60	(11.79,15.41)
Jamalpur	8,471	15,218	1,535	146	65	57	8	9.59	(8.05,11.14)	4.27	(3.24,5.31)	13.87	(12.01,15.72)
Jangal	4,683	8,581	653	54	19	19	0	6.29	(4.62,7.97)	2.21	(1.22,3.21)	8.51	(6.56,10.45)
Narua	7,005	12,612	1,180	114	24	20	4	9.04	(7.39,10.69)	1.90	(1.14,2.66)	10.94	(9.13,12.76)
Nawabpur	11,010	19,989	1,903	193	53	51	2	9.66	(8.3,11.01)	2.65	(1.94,3.36)	12.31	(10.78,13.84)

Table S2. Proportion of pregnancy loss, spontaneous abortion, and induced abortion among pregnant women

Variables	pregnant women (LMP between Aug 1, 21, and Nov 30, 22)	Women experiencing miscarriage/ spontaneous abortion	Women experiencing induced abortion	Women experiencing miscarriage/ spontaneous abortion or Induced abortion	Miscarriage /spontaneous abortion	95% CI	Induced abortion	95% CI	Miscarriage/ spontaneous abortion or induced abortion)	95% CI
	n	n	n	n	%		%		%	
**Total**	**7612**	**803**	**240**	**1043**	**10.55**	**(9.86,11.24)**	**3.15**	**(2.76,3.55)**	**13.70**	**(12.93,14.47)**
**Age of woman**										
15-19	2286	218	42	260	9.54	(8.33,10.74)	1.84	(1.29,2.39)	11.37	(10.07,12.68)
20-24	2309	215	51	266	9.31	(8.13,10.5)	2.21	(1.61,2.81)	11.52	(10.22,12.82)
25-29	1658	182	42	224	10.98	(9.47,12.48)	2.53	(1.78,3.29)	13.51	(11.86,15.16)
30-34	972	132	59	191	13.58	(11.43,15.73)	6.07	(4.57,7.57)	19.65	(17.15,22.15)
35+	387	56	46	102	14.47	(10.97,17.98)	11.89	(8.66,15.11)	26.36	(21.97,30.75)
**Age of Husband**										
15-19	371	31	10	41	8.36	(5.54,11.17)	2.70	(1.05,4.34)	11.05	(7.86,14.24)
20-24	1256	138	20	158	10.99	(9.26,12.72)	1.59	(0.9,2.28)	12.58	(10.75,14.41)
25-29	1512	161	26	187	10.65	(9.09,12.2)	1.72	(1.06,2.37)	12.37	(10.71,14.03)
30-34	1736	167	60	227	9.62	(8.23,11.01)	3.46	(2.6,4.32)	13.08	(11.49,14.66)
35+	2737	251	108	359	9.17	(8.09,10.25)	3.95	(3.22,4.68)	13.12	(11.85,14.38)
missing	719	55	16	71						
**Education of the woman**										
No education	194	18	20	38	9.28	(5.2,13.36)	10.31	(6.03,14.59)	19.59	(14,25.17)
Primary incomplete	532	64	23	87	12.03	(9.27,14.79)	4.32	(2.6,6.05)	16.35	(13.21,19.5)
Primary complete	537	61	36	97	11.36	(8.68,14.04)	6.70	(4.59,8.82)	18.06	(14.81,21.32)
Secondary incomplete	3971	416	126	542	10.48	(9.52,11.43)	3.17	(2.63,3.72)	13.65	(12.58,14.72)
Secondary complete or higher	2378	244	35	279	10.26	(9.04,11.48)	1.47	(0.99,1.96)	11.73	(10.44,13.03)
**Education of the husband**										
No education	864	101	54	155	11.69	(9.55,13.83)	6.25	(4.64,7.86)	17.94	(15.38,20.5)
Primary incomplete	1,343	126	51	177	9.38	(7.82,10.94)	3.80	(2.78,4.82)	13.18	(11.37,14.99)
Primary complete	915	101	25	126	11.04	(9.01,13.07)	2.73	(1.68,3.79)	13.77	(11.54,16)
Secondary incomplete	1,874	209	60	269	11.15	(9.73,12.58)	3.20	(2.4,4)	14.35	(12.77,15.94)
Secondary complete or higher	1,894	211	34	245	11.14	(9.72,12.56)	1.80	(1.2,2.39)	12.94	(11.42,14.45)
missing	722	55	16	71						
**Education of the maternal grandmother**										
No education	782	73	10	83	9.34	(7.3,11.37)	1.28	(0.49,2.07)	10.61	(8.45,12.77)
Primary incomplete	353	35	8	43	9.92	(6.8,13.03)	2.27	(0.71,3.82)	12.18	(8.77,15.59)
Primary complete	242	24	3	27	9.92	(6.15,13.68)	1.24	(-0.15,2.63)	11.16	(7.19,15.12)
Secondary incomplete	331	29	7	36	8.76	(5.72,11.81)	2.11	(0.56,3.66)	10.88	(7.52,14.23)
Secondary complete or higher	25	2	0	2	8.00	(-2.63,18.63)	0.00	(0,0)	8.00	(-2.63,18.63)
missing	5,879	640	212	852						

**Profession of woman**										
Not involved in income-generating activity	7,257	752	221	973	10.36	(9.66,11.06)	3.05	(2.65,3.44)	13.41	(12.62,14.19)
Involved in income-generating activity	355	51	19	70	14.37	(10.72,18.01)	5.35	(3.01,7.69)	19.72	(15.58,23.86)
**Profession of Husband**				0						
Not involved in income-generating activity	551	63	7	70	11.43	(8.78,14.09)	1.27	(0.34,2.21)	12.70	(9.92,15.48)
Involved in income-generating activity	6,342	685	217	902	10.80	(10.04,11.56)	3.42	(2.97,3.87)	14.22	(13.36,15.08)
missing	719	55	16	71						
**Wealth quintile**										
Lowest	1,289	120	42	162	9.31	(7.72,10.9)	3.26	(2.29,4.23)	12.57	(10.76,14.38)
Second	1,419	153	60	213	10.78	(9.17,12.4)	4.23	(3.18,5.28)	15.01	(13.15,16.87)
Middle	1,572	180	47	227	11.45	(9.88,13.02)	2.99	(2.15,3.83)	14.44	(12.7,16.18)
Fourth	1,748	194	50	244	11.10	(9.63,12.57)	2.86	(2.08,3.64)	13.96	(12.33,15.58)
Highest	1,584	156	41	197	9.85	(8.38,11.32)	2.59	(1.81,3.37)	12.44	(10.81,14.06)
**Family size**										
≤4	4,185	494	119	613	11.80	(10.83,12.78)	2.84	(2.34,3.35)	14.65	(13.58,15.72)
≥5	3,427	309	121	430	9.02	(8.06,9.98)	3.53	(2.91,4.15)	12.55	(11.44,13.66)
**Number of children**										
0	703	323	15	338	45.95	(42.26,49.63)	2.13	(1.07,3.2)	48.08	(44.39,51.77)
1	2,734	248	58	306	9.07	(7.99,10.15)	2.12	(1.58,2.66)	11.19	(10.01,12.37)
2	2,439	158	89	247	6.48	(5.5,7.45)	3.65	(2.9,4.39)	10.13	(8.93,11.32)
>=3	1,732	74	78	152	4.27	(3.32,5.22)	4.50	(3.53,5.48)	8.78	(7.44,10.11)
missing	4	-	-	-	-	-	-	-	-	-
**History of pregnancy loss**										
No	6,164	619	168	787	10.04	(7.96,12.12)	2.73	(2.32,3.13)	12.77	(11.93,13.6)
Yes	1,448	184	72	256	12.71	(10.4,15.01)	4.97	(3.85,6.09)	17.68	(15.71,19.64)
**History of stillbirth**										
No	7,336	769	225	994	10.48	(8.36,12.6)	3.07	(2.67,3.46)	13.55	(12.77,14.33)
Yes	276	34	15	49	12.32	(10.05,14.59)	5.43	(2.76,8.11)	17.75	(13.25,22.26)
**Union**										
Baharpur	1,280	166	40	206	12.97	(10.65,15.29)	3.13	(2.17,4.08)	16.09	(14.08,18.11)
Baliakandi	1,051	104	32	136	9.90	(7.83,11.96)	3.04	(2.01,4.08)	12.94	(10.91,14.97)
Islampur	1,178	122	49	171	10.36	(8.25,12.46)	4.16	(3.02,5.3)	14.52	(12.5,16.53)
Jamalpur	1,146	117	50	167	10.21	(8.12,12.3)	4.36	(3.18,5.55)	14.57	(12.53,16.62)
Jangal	481	38	17	55	7.90	(6.03,9.77)	3.53	(1.88,5.18)	11.43	(8.59,14.28)
Narua	977	97	16	113	9.93	(7.86,12)	1.64	(0.84,2.43)	11.57	(9.56,13.57)
Nawabpur	1,499	159	36	195	10.61	(8.48,12.74)	2.40	(1.63,3.18)	13.01	(11.31,14.71)

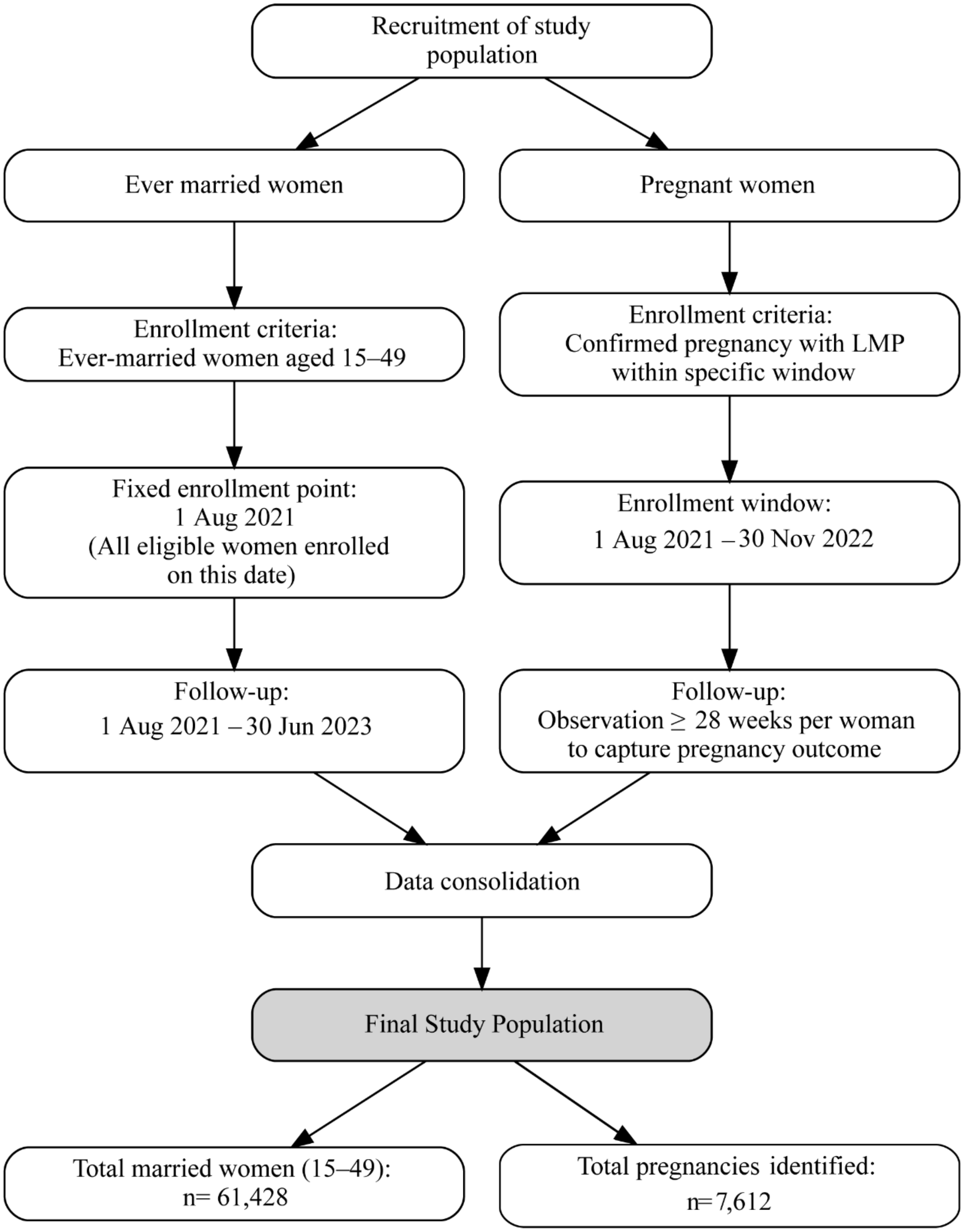

Figure S1. Selection of the study sample.
